# Fragility Fractures of the Femur in Spinal Cord Injury Patients

**DOI:** 10.7759/cureus.90561

**Published:** 2025-08-20

**Authors:** Georgios E Prelorentzos, Andreas Artinopoulos, Ioannis S Benetos, Angelos Kaspiris, John Vlamis

**Affiliations:** 1 4th Department of Orthopaedic Surgery, National and Kapodistrian University of Athens (NKUA) KAT Attica General Hospital, Athens, GRC; 2 3rd Department of Orthopaedic Surgery, National and Kapodistrian University of Athens (NKUA) KAT Attica General Hospital, Athens, GRC

**Keywords:** femoral fracture, fragility fracture, osteoporosis, paraplegia, spinal cord injury, tetraplegia

## Abstract

Individuals with spinal cord injury (SCI) experience rapid and profound bone loss below the level of injury, predisposing them to fragility fractures. Due to their frequency and clinical impact, femoral fragility fractures in SCI patients require special consideration. This literature review aims to summarize the current evidence on the epidemiology, pathophysiology, diagnosis, management, and prevention of femoral fragility fractures in SCI patients.

A comprehensive literature search was conducted in Medical Literature Analysis and Retrieval System Online (MEDLINE) and Cumulative Index to Nursing and Allied Health Literature (CINAHL) databases using the Preferred Reporting Items for Systematic Reviews and Meta-Analyses (PRISMA) 2020 guidelines. Studies focusing on femoral fractures in adults with SCI were included, while reviews, pediatric studies, animal studies, and non-English publications were excluded.

Femoral fractures account for up to 61% of lower limb fractures in the SCI population, with the distal femur and hip being the most common sites. Risk factors include age, time since injury, motor-complete injuries, and low bone mineral density (BMD). Loss of BMD occurs rapidly after SCI and plateaus two to five years post-injury. Clinical presentation may be subtle, often requiring high suspicion and imaging. Operative management is favored over conservative approaches due to lower complication and non-union rates. Preventive strategies include early BMD assessment, vitamin D and calcium optimization, weight-bearing therapy, and pharmacologic agents such as bisphosphonates and denosumab.

Femoral fragility fractures are among the most common and clinically significant complications in patients with SCI. Timely diagnosis, appropriate surgical management, and targeted prevention strategies are critical to reducing complications and maintaining function. There is a need for consistent screening protocols and further research to optimize care in this vulnerable population.

## Introduction and background

In most European countries, the annual incidence of traumatic spinal cord injuries (SCI) is uniformly around 20 cases per million people, with few exceptions [1]. Despite the varying reported incidence and the selection bias regarding non-traumatic SCI, there is a shift in the epidemiology of SCI, with a study reporting that non-traumatic made up 61% of total SCI treated in a rehabilitation clinic [2]. Regardless of the initial cause, these are incredibly debilitating injuries that have a cost on multiple aspects of patients’ health.

It has been previously well documented that the bone mineral density (BMD) of patients with SCI decreases rapidly below the level of injury, with a rate of 1% per week, within the first months from injury [3]. Sublesional osteoporosis is multifactorial, and the processes involved in its development include alterations in the mechanical loading of bone, bone remodeling, and blood flow, as well as the nutritional and hormonal status of the patient. The rapid loss of BMD predisposes to fractures caused by low-energy trauma that significantly impact the morbidity and mortality of patients with SCI. These fragility fractures can affect all appendages but are more commonly located in the lower extremity, with a study reporting that 83% of long-bone fractures involve the lower limb [4]. According to a recent study, 25% to 46% of people with chronic SCI will suffer a lower extremity fracture during their lifetime [5].

Fractures of the femur account for 61% of all fragility fractures in this population, and besides the fracture-related complications, they also restrict the patient from using mobility assistive devices such as passive standing frames, exoskeletons, and bed-to-wheelchair self-transfer [6]. Their treatment has been a topic of controversy due to the unique profile of the SCI patient. In the past, conservative treatment was preferred for these patients, but it was strongly correlated with non-union, malunion, and pressure ulcers [7, 8]. When considering surgical treatment, the treating surgeon should take into account the risks and benefits associated with surgery and the patient’s pre-injury functional level, mobility, and independence, along with possible use of future mobility technology [9].

To the best of our knowledge, there are no published reviews summarizing results and focusing on fragility fractures of the femur in SCI patients yet. This review aims to summarize current evidence on the epidemiology, pathophysiology, and diagnosis of femoral fragility fractures in patients with SCI and discuss different treatment strategies.

## Review

Materials and methods

**Figure 1:**
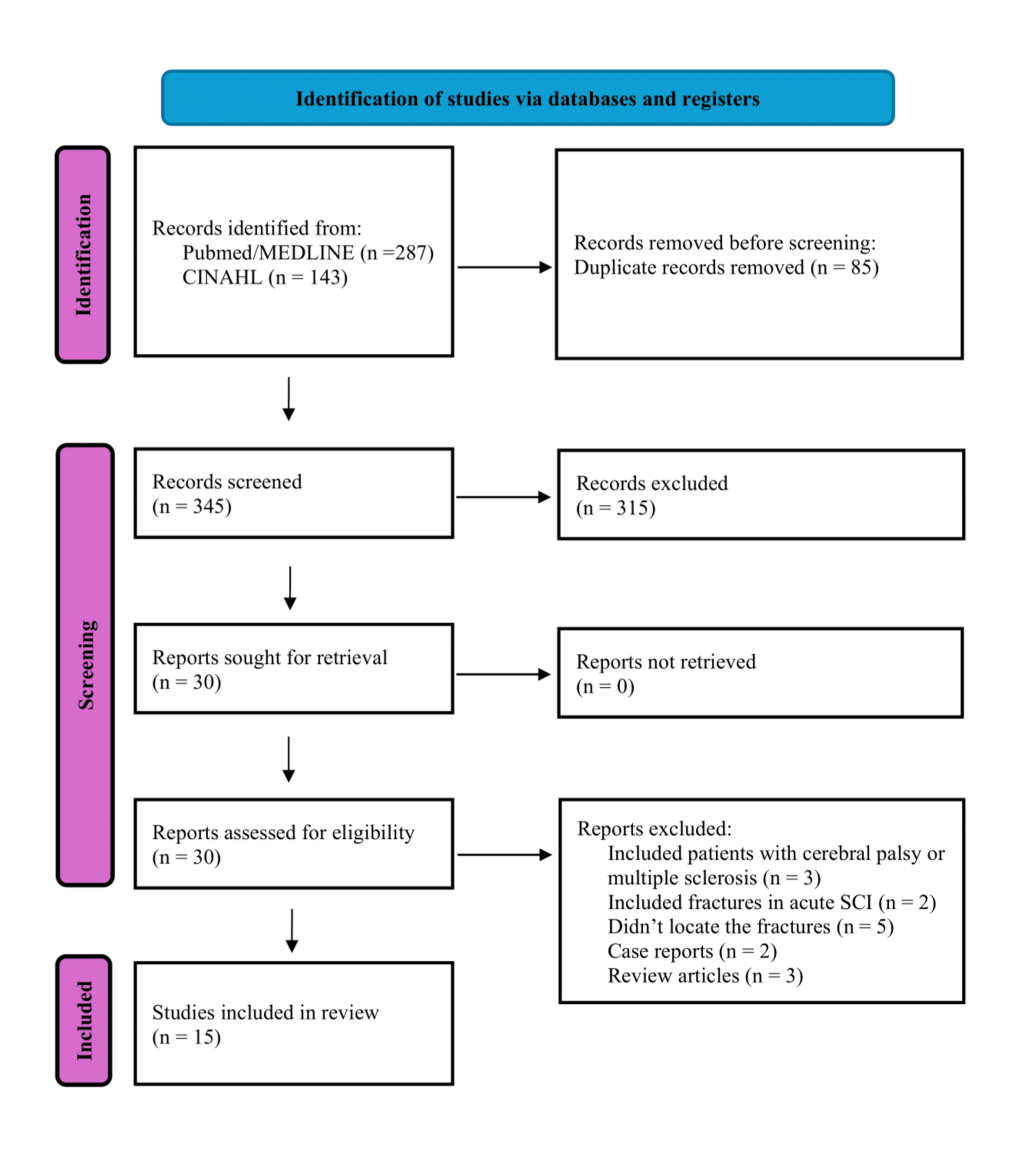
PRISMA flowchart depicting article screening and selection PRISMA: Preferred Reporting Items for Systematic Reviews and Meta-Analyses; MEDLINE: Medical Literature Analysis and Retrieval System Online (MEDLINE); CINAHL: Cumulative Index to Nursing and Allied Health Literature; SCI: spinal cord injury [10]

A comprehensive search of literature was conducted across Medical Literature Analysis and Retrieval System Online (MEDLINE) and Cumulative Index to Nursing and Allied Health Literature (CINAHL) electronic databases according to the updated Preferred Reporting Items for Systematic Reviews and Meta-Analyses (PRISMA) 2020 guidelines [10]. The search targeted peer-reviewed articles related to femoral fractures in individuals with SCI or related conditions such as paraplegia and tetraplegia that were published from January 1, 2000, until May 31, 2025. A combination of Medical Subject Headings (MeSH) and free-text keywords (Title/Abstract) was used to increase the sensitivity and specificity of the search. The search terms included "femoral fracture," "femur fracture," "hip fracture," "lower extremity fracture," "fragility fracture," "spinal cord injury," "SCI," "paraplegia," and "tetraplegia." The final search was completed on June 5, 2025. Non-English articles, animal studies, review articles, studies on pediatric populations, and studies unrelated to SCI or fracture location were excluded. Titles and abstracts were reviewed independently by two reviewers, who also evaluated the full texts for eligibility. Disagreements were settled through discussion or adjudicated by a third author. The initial search yielded 430 results, and 85 duplicate records were removed. The remaining 345 titles and abstracts were screened, and 315 studies failed to meet the inclusion criteria. The remaining 30 full-text articles were retrieved. Fifteen reports were excluded from the review because they included patients with cerebral palsy or multiple sclerosis (n=3), they included fractures in acute SCI (n=2), they didn’t locate the fractures (n=5), they were case reports (n=2), or they were review articles (n=3). The selection process can be visualized in the study flowchart (Figure 1).

Results

A total of 15 studies met the selection criteria. Of these, 12 were retrospective in design, while three were cross-sectional studies. Notably, no prospective studies were identified. The largest patient cohorts were reported in retrospective studies that analyzed data from veteran health registries. Most studies (n=9) focused on evaluating the outcomes of various management strategies for fragility fractures in individuals with SCI. Their findings are summarized in Table 1. A smaller subset of studies (n=6) investigated potential risk factors associated with fragility fractures and explored the circumstances around them. Their findings are summarized in Table 2.

**Table 1:** Studies that evaluated management strategies for fragility fractures in SCI patients SCI: spinal cord injury; RCS: retrospective cohort study; CSS: cross-sectional study; LE: lower extremity

Author	Study Design	Number of SCI patients	Intervention	Results
Martínez et al., 2002 [8]	RCS	26	17 LE fractures treated operatively, 9 LE fractures treated nonoperatively	Union was achieved in all cases, regardless of treatment; a high rate of pressure sores in patients treated nonoperatively.
Meiners et al., 2003 [11]	RCS	21	22 LE fractures treated with external ring fixator	Retained mobility of the joints, relatively low complication rate, and long average hospital stay.
Bishop et al., 2013 [7]	RCS	396	148 femoral fractures treated operatively, 248 femoral fractures treated nonoperatively	Surgical treatment in SCI patients was not associated with higher mortality or complications compared to the general population; nonoperative SCI patients experienced more adverse events (29% vs. 21%).
Uehara et al., 2013 [12]	RCS	15	20 LE fractures treated with a soft plastic brace	High rate of angular deformities; long average hospital stay.
Frotzler et al., 2015 [6]	RCS	107	130 LE fractures treated operatively, 26 LE fractures treated nonoperatively	SCI was correlated with simple or extra-articular fractures of the distal femur; the fracture-related complication rate was comparable between the two groups.
Bethel et al., 2015 [4]	RCS	12162	77 femoral fractures treated operatively, 595 femoral fractures treated nonoperatively	A minority of patients received surgical treatment. Hip fractures were the most common fracture site treated with surgery (13.2%), followed by fractures of the femur (10.7%).
Grassner et al., 2018 [13]	RCS	132	83 LE fractures treated operatively, 49 LE fractures treated nonoperatively	Higher non-union rate in the nonoperative group (22.5% vs 12%); longer time since SCI, delay in treatment, femur fracture, and fracture category (B) were associated with increased non-union rates.
Fouasson et al. 2019 [14]	RCS	40	40 LE fractures treated operatively, 19 LE fractures treated nonoperatively	25.0% of surgically managed fractures had at least one medical or post-surgical complication compared to 63.2% of the non-operative group.
Ung et al., 2019 [15]	RCS	58	58 LE fractures treated operatively: 41 with internal fixation, 17 with external fixation	High non-union rate regardless of osteosynthesis method; more surgery-related complications in the internal fixation group.

**Table 2:** Studies that investigated risk factors for fragility fractures in SCI patients SCI: spinal cord injury; RCS: retrospective cohort study; CSS: cross-sectional study; BMD: bone mineral density; QCT: quantitative computed tomography; pQCT: peripheral quantitative computed tomography; DXA: dual-energy X-ray absorptiometry; ASIA: American Spinal Injury Association

Author	Study design	Number of SCI patients	Key findings
Zehnder et al., 2004 [16]	CSS	100	The total number of individual fractures and the frequency of fracture episodes increased progressively with time since SCI. Bone loss after SCI is a process restricted to unloaded skeletal sites.
Eser et al., 2005 [3]	CSS	99	Trabecular BMD of the epiphyses is the most sensitive parameter to assess fracture risk in SCI patients. It is better evaluated by QCT or pQCT because with DXA, the trabecular bone compartment cannot be separated from the thin cortical shell on the bone’s perimeter.
Lala et al. 2014 [17]	CSS	70	Motor complete injury (ASIA A-B) and BMD were significantly associated with fragility fractures
Akhigbe et al., 2015 [18]	RCS	140	Wheelchair and transfer-related activities were highly associated with fragility fractures and should be a focus for the prevention of fractures in SCI patients
Bethel et al., 2016 [19]	RCS	12162	Injury completeness increased the risk for fracture in tetraplegics but not in paraplegics
Champs et al., 2020 [5]	RCS	325	The femur was the most common fracture location in wheelchair-bound SCI patients, but not in ambulatory individuals

Discussion

Epidemiology

A study in 100 paraplegic men reported a mean overall fracture incidence of 2.2%. In the same study, it was highlighted that the time from the initial SCI was an important risk factor that increased the fracture incidence to 4.6% for patients living with SCI ranging from 20 to 29 years [16]. In a large observational registry study on 12,162 male veterans, 1,634 lower limb fractures were identified. When considering both hip and shaft/distal femoral fractures, the femur was the most frequently fractured bone, accounting for 41.1% of those fractures [4]. The majority of studies that reported on the location of fractures on SCI patients showed similar results, with tibial fractures following in frequency [3,5,6,8,12-15,17,19]. In the referenced bibliography, the localization of femoral fractures varied a lot. Some researchers discerned between proximal, shaft, and distal femur fractures, while others only between hip/proximal and shaft/distal femur. In the largest among these studies, Bethel et al. [4] found that in a total of 672 fractures of the femur, hip fractures accounted for 253 (37.6%). Hip fractures were further categorized as femoral neck (n=169, 25.1%), trochanteric (n=18, 2.7%), intertrochanteric (n=10, 1.5%), and subtrochanteric (n=56, 8.3%). On the contrary, the authors did not discern between shaft and distal femur fractures. In the review by Bishop et al. [7] on the outcomes of surgical vs. nonsurgical management of femoral fractures in SCI patients, 49% of fractures involved the proximal femur, while 51% involved the shaft or distal femur. Interestingly, when the researchers compared their results with the general population, the same percentages were 93% for the proximal and 7% for the shaft/distal femur. In five studies that discerned between proximal, shaft, and distal femur, it was shown that the most common location for fractures was the distal part of the femur, followed by the proximal and the shaft [3, 8, 13, 14, 16]. Four publications further categorized hip and proximal femur fractures. In two small studies, fractures were distributed homogeneously between femoral neck, intertrochanteric, and subtrochanteric [8, 14]. Bethel et al. [4] found a higher prevalence of femoral neck and subtrochanteric fractures, while Bishop et al. [7] found that intertrochanteric fractures accounted for more than half of hip fractures. Only two studies further characterized fractures of the distal femur, with the majority of them being in the supracondylar region [14, 16].

In the one study that included solely fractures of the femur, it was shown that the SCI population sustained these fractures at a younger age than the general population (mean age 60 years vs. 74 years) [7]. A multitude of studies reported on the mean age of lower extremity fracture occurrence, which ranged from 45 to 57 years old, along with the mean time between SCI and fracture, which ranged from nine to 20 years [5, 6, 8, 11-14, 17]. In summary, the literature consistently identifies the distal femur as the predominant location of fragility fractures in SCI patients, with a higher frequency observed in those with prolonged injury duration.

Risk Factors

Several risk factors for fracture have been identified in the SCI population. The risk rises with increasing age and time post injury [20]. Patients with paraplegia and patients with motor complete injuries (American Spinal Injury Association (AIS) A-B) are at greater risk for fragility fractures [17,19,21]. The decrease of BMD at the distal femur and proximal tibia predisposes to fractures and is a significant predictor of their number [3,20,22]. Notably, men and women with SCI have been found to sustain fractures with comparable frequency [19]. This comes in contrast with the general osteoporotic population, where women appear to be at increased risk for fragility fractures compared to men [23]. Across the included studies, no risk factors were identified that predicted specific fracture locations or patterns. In 2023, Craven et al. [24] developed and published the Spinal Cord Injury-Fracture (SCI-FX) tool, a risk assessment tool that estimates five-year lower limb fracture risk in patients living with chronic SCI. The SCI-FX tool is not yet validated in an independent cohort. It is currently a preliminary risk model that requires formal external validation before it can be confidently used in clinical practice.

Pathophysiology

The basis of the sublesional osteoporosis pathophysiology lies in the mechanical unloading due to the neurologic deficit of the SCI patient. Sclerostin is an important bone formation inhibitor, produced by osteocytes, that is upregulated by mechanical unloading [25]. Sclerostin works indirectly in two ways. Its anti-anabolic function is caused by downregulating the Wnt/β-catenin pathway in osteoblasts, while its catabolic function is caused by upregulating receptor activator of nuclear factor kappa-Β ligand (RANKL) and downregulating osteoprotegerin (OPG) excretion by osteocytes [25]. The latter leads to increased osteoclast differentiation and bone resorption. The study by Battaglino et al. found that serum sclerostin levels increased in the acute and short-term SCI (<5 years post SCI) and then decreased with the chronicity of the disease, proposing that sclerostin can be used as a marker of osteoporosis severity in SCI [26]. Hormonal changes and adipose tissue accumulation also influence the bone homeostasis in these patients, but the exact mechanism has yet to be defined.

A histomorphometry-based study showed that in the acute setting and the first months after SCI, there was decreased osteoblastic and increased osteoclastic activity [27]. This phenomenon reached a steady state between two and seven years after injury, and it is in accordance with the research done by Battaglino et al. [26]. The imbalance in osteoblastic and osteoclastic activity is typically followed and supported by hypercalciuria, hypercalcemia, and increased levels of bone resorption markers like N-telopeptide (NTX) and C-telopeptide (CTX) [28].

In the first months after SCI, the patient loses BMD rapidly until a new steady state is reached. BMD around the knee decreases at a rate of 1% every week in the first months since injury, reaching a total 50% reduction two years after SCI [3, 29, 30]. Studies reporting on the new steady state found that it is reached two to five years from injury [3, 31]. The loss of BMD affects predominantly the hip, the distal femur, and the proximal tibia. The BMD of the spine remains mostly unaffected, regardless of the time since injury, and this finding can be attributed to the weight that is distributed through the spine when SCI patients sit or mobilize with assistive devices [31].

Clinical Presentation and Diagnosis

Depending on the level and severity of SCI (ASIA Impairment Scale), a femoral fracture might present with pain or be asymptomatic due to a lack of pain sensation. In that case, healthcare providers and patients should be vigilant for signs like gross motion at the fracture site, spasticity due to reported pain, and inability to transfer. A femoral fracture will also compromise skeletal stability and will limit the patient from using mobility assistive devices and passive standing frames. The majority of lower extremity fractures occur during wheelchair use or transfers, and there should be a high level of suspicion after incidents like falls and collisions [14, 18]. Additionally, in patients with SCI at the level of T6 or above, fractures of the femur may first present themselves with autonomic dysreflexia, a life-threatening emergency [32].

Femoral fractures have been reported to occur from minor trauma or spontaneous insufficiency, and they frequently go unreported [33]. Delayed diagnosis may lead to further displacement and comminution due to the continuation of transfers and passive movements, potentially rendering a manageable fracture into a surgical challenge. Interestingly, the delay of hospitalization in an adequate center has been associated with higher rates of non-union [13]. A delayed diagnosis further increases the risk for secondary complications like deep vein thrombosis (DVT) and pressure ulcers. Furthermore, owing to the compromised skeletal stability, an undiagnosed fracture of the femur will prolong the bed rest of the patient, leading to deconditioning and joint contractures, ultimately limiting independence and quality of life.

After careful clinical examination, X-rays are usually sufficient for fracture diagnosis. A computed tomography (CT) scan is invaluable in cases of highly comminuted or intra-articular fractures, where operative management is indicated. Magnetic resonance imaging (MRI) can be useful in diagnosing occult fractures of the hip [34].

Management

In the past, nonoperative treatment was the treatment of choice for the management of lower limb fractures in patients living with chronic SCI, mostly because of concern about surgical complications [35]. This included the use of pillows, soft splints, and traction, usually combined with bed rest. However, in multiple studies, nonoperative treatment was correlated with an increased rate of complications and prolonged hospital stay [7, 12]. The most common complications associated with conservative treatment are pressure ulcers, malalignment, and non-union [7, 12-14]. In their study, Bethel et al. [4] reported high rates of amputations that were attributed to unsuccessful nonoperative treatments. Currently, there is a clear trend favoring the operative management of long bone fractures in the SCI population, and femoral fractures are vastly the most frequently treated with surgery [4, 13, 14]. Locking compression plates, intramedullary nailing, external fixation, and arthroplasty are all valid surgical options for such fractures, but surgeons should take great care with each of these, implementing extreme bone loss principles. Frotzler et al. [6] found that femoral fractures were mainly treated with locking compression plates. In a large retrospective cohort, there was no difference in the rate of complications for surgically treated femoral fractures between SCI patients and the general population [7]. Infection, wound healing issues, and hematoma are the most common adverse events after operative management [14].

In 2022, the clinical practice guidelines (CPG) for acute lower extremity fracture management in chronic SCI were published [9]. The authors recommended operative management for open fractures, fractures that are prone to healing with malalignment, and fractures where nonoperative treatment failed. They advocated that treatment decisions should be made based on the patient's preinjury level of function, preferences, and occupation while always optimizing the use of current and future mobility technologies. Internal fixation with a plate or intramedullary nailing and arthroplasty were recommended for hip and femoral fractures, but no indications for external fixation were included. Resection arthroplasty is an option for displaced fractures of the femoral head, but it can lead to proximal migration of the femur and subsequent pressure ulcer or sitting imbalance.

Surgical technique should be dictated by the principles of osteoporotic fracture fixation, utilizing developments like bone augmentation, bone impaction, wide buttress fixation, and lever arm modification [36]. Increased stability constructs like double plating and nail-plate fixation are invaluable when treating distal femoral fractures where bone loss and comminution are involved [37]. Most SCI patients lack sensorimotor feedback of the lower limb, causing a higher load on the osteosynthetic material due to a longer biomechanical momentum. Using longer implants can help achieve more stable osteosynthesis and effectively manage this issue [15]. In the study by Grassner et al. [13], all Garden III and IV femoral neck fractures showed non-union regardless of treatment received. Taking this finding into consideration, displaced femoral neck fractures in SCI patients should be predominantly treated with arthroplasty. When arthroplasty is considered, cemented fixation of the femoral stem will lower the incidence of periprosthetic fractures during wheelchair transfers [38]. Lastly, in the CPG, the role of physical and occupational therapists in post-fracture rehabilitation was highlighted, recommending their prompt involvement and assistance in weight bearing, range of motion, and transfer restrictions [9].

Prevention Strategies

Femoral fractures greatly diminish the quality of life of chronic SCI patients by delaying rehabilitation, limiting mobility, and impeding vocation and avocation. The prevention of fractures should be a priority for the multidisciplinary treating team promptly after the acute phase of injury. Screening for sublesional osteoporosis includes medical history, risk factor assessment, laboratory testing for secondary osteoporosis (e.g., vitamin D deficiency), and diagnostic imaging modalities [39]. According to the International Society of Clinical Densitometry (ISCD) guidelines, dual-energy X-ray absorptiometry (DXA) is the gold standard for assessing bone density in SCI patients. Scans of the total hip, distal femur, and proximal tibia should be used to predict fracture risk and monitor response to therapy [40]. Antoniou et al. [31] proposed that initial BMD evaluation should start as early as six months post SCI, with regular re-evaluation every 12 months. The diagnosis of osteoporosis is made by calculating T-scores and Z-scores according to the patient’s age or with a fragility fracture, irrespective of the BMD measured [39].

In all SCI patients, it is wise to optimize calcium intake and vitamin D supplementation to ensure skeletal health [39]. The above can be complemented with weight-bearing therapy like passive standing and walking training. Assistive devices, including standing frames, standing wheelchairs, long leg braces, treadmills, and exoskeletons, can be used. Weight-bearing activities have been proven to reduce BMD decline when incorporated in SCI rehabilitation [41]. Functional electrical stimulation (FES) is another modality with established benefits on bone health that could be included in the patient’s rehabilitation regimen [42]. Although weight-bearing therapy and FES have been shown to improve BMD in SCI patients, patient adherence can be a significant limiting factor, as these interventions require sustained effort, motivation, and access to specialized equipment.

Treatment should be initiated as soon as a diagnosis of osteoporosis is made for the SCI patient. Pharmacological management usually involves bisphosphonates. Bisphosphonates are approved by the Food and Drug Administration (FDA) for the treatment of osteoporosis in the general population, including postmenopausal women and men at high risk for fractures. However, their use in SCI-related osteoporosis is considered off-label, though supported by evidence. Alendronate (orally once per week) and zoledronic acid (one-time intravenous infusion) are bisphosphonates studied widely on the SCI population, with proven efficacy on BMD [43, 44]. Furthermore, denosumab is a recent advancement, initially approved for use in postmenopausal women, but emerging evidence indicates its effectiveness in increasing BMD around the knee and hip [45, 46].

Limitations

This review has several limitations. All included studies were retrospective or cross-sectional, which may introduce bias and limit the ability to establish causality. Additionally, a formal quality assessment of the included studies was not performed, which could affect the overall strength of the evidence presented. Finally, the heterogeneity in study populations, fracture definitions, and outcome measures may limit the generalizability of the findings. Despite these limitations, the review provides a comprehensive overview of fragility fractures in SCI patients and underscores the need for more rigorous, prospective research.

## Conclusions

The importance of timely screening and osteoporosis treatment initiation cannot be understated. Even though there have been suggestions for when to perform the first DXA scan post-injury, there are currently no universally implemented guidelines. When it comes to prevention, educating patients and their caregivers on recognizing potential fracture symptoms and practicing safe wheelchair transfers should be a priority for SCI centers. All patients living with chronic SCI are at risk of fragility fractures, and those of the femur are the most common. It has been well-established that operative treatment is preferred for these fractures and that the risk of surgical complications is comparable to that of the general population. Longer, high-stability fixation constructs spanning the whole femur should always be considered for femoral fractures complicated by bone loss and comminution. They permit immediate weight-bearing and use of assistive mobility devices, while simultaneously reducing the risk of future periprosthetic fractures and implant failure. Future research should focus on prospective, multicenter, and randomized controlled studies to strengthen the evidence base for fracture prevention and management in SCI patients. Furthermore, validation of the SCI-FX risk score is needed to enhance fracture prediction and support individualized clinical decision-making.
